# Editorial: 10 years of Frontiers in genetics: past discoveries, current challenges and future perspectives

**DOI:** 10.3389/fgene.2023.1192071

**Published:** 2023-05-05

**Authors:** William C. Cho, Jordi Pérez-Tur, Rosalba Giugno, Mehdi Pirooznia, Kathleen Boris-Lawrie, Dov Greenbaum, Mojgan Rastegar, Rui Henrique, Peng Xu, Joao Batista Teixeira da Rocha, Blanka Rogina

**Affiliations:** ^1^ Queen Elizabeth Hospital, Kowloon, Hong Kong SAR, China; ^2^ Institut de Biomedicina de València-CSIC, CIBER-CIBERNED, ISCIII, Valencia, Spain; ^3^ Department of Computer Science, University of Verona, Verona, Italy; ^4^ Johnson & Johnson, Washington, DC, United States; ^5^ Department of Veterinary and Biomedical Sciences, University of Minnesota Twin Cities, St. Paul, MN, United States; ^6^ Department of Molecular Biophysics and Biochemistry, Yale University, New Haven, CT, United States; ^7^ Zvi Meitar Institute for Legal Implications of Emerging Technologies, Reichman University, Herzliya, Israel; ^8^ Department of Biochemistry and Medical Genetics, Max Rady College of Medicine, Rady Faculty of Health Sciences, University of Manitoba, Winnipeg, MB, Canada; ^9^ Portuguese Oncology Institute of Porto (IPO Porto)/Porto Comprehensive Cancer Center (Por-to.CCC), R. Dr. António Bernardino de Almeida, Porto, Portugal; ^10^ School of Medicine and Biomedical Sciences (ICBAS), University of Porto (ICBAS-UP), Porto, Portugal; ^11^ Xiamen University, Xiamen, China; ^12^ Departamento de Bioquímica e Biologia Molecular (UFSM), Federal University de Santa Maria, Santa Maria, Brazil; ^13^ Department of Genetics and Genome Sciences, School of Medicine, University of Connecticut Health Center, Farmington, CT, United States

**Keywords:** genetics, genomics, Frontiers in genetics, human physiology, genomics data analysis

This Editorial is for the Research Topic dedicated to 10 years of Frontiers in Genetics. Frontiers in Genetics was launched in 2011 with the grand challenge proposed by Professor David Allison, founding Field Editor, to use new and exciting technologies—such as the mathematical and biological tools of modern genomics—to answer some of the key questions about evolutionary biological science. The second challenge was designed to address genomic structure, tissue-specific gene expression during development, aging, and disease. The final challenge was aimed at understanding how modification of gene expression could affect health and longevity using modern genomic tools such as knockout mice, transgenic plants or gene therapies.

The answers to these three challenges have resulted in the publication of leading edge genetics and genomic studies, ranging from basic to translational research, perspectives, and commentaries. Ten years later, Frontiers in Genetics has arguably accomplished its initial stated goals, and has even expanded remarkably, maturing into a platform to publish new discoveries, views, perspectives, advances and challenges in the field, ultimately becoming the largest open access journal in genetics.

In 2011, Frontiers in Genetics had twelve mother-field Specialty Sections. Today it has 23 Specialty sections, each directed by dedicated Chief Editors, as well as an outstanding editorial board of over 7,500 dedicated and exceptional Associated Editors and Reviewers. During our first 10 years, Frontiers in Genetics has collectively published over 6,725 articles, all of which are freely available online to scientists, doctors, patients, policymakers and the general public. Some exceptional statistics: The current impact factor is 4.772, which keeps on rising, and the most cited manuscript, with over 502 citations, is by Sawana et al. (2014), which describes the molecular signature and phylogenomic analysis of the genus Burkholderia. There were overall of 1,12,857 articles citations between 2011 and 2021 and 182 articles were cited more than 100 times. There was an impressive number of total downloads of 1,15,36,824, with a perspective on the origins of the Ashkenaz, Ashkenazic Jews and Yiddish leading this impressive record with 4,34,504 views (Das et al., 2017). Lastly, there were 676 Research Topics posted between 2011 and 2021.

This Research Topic is a celebration of the 10 year Anniversary of this remarkable journal, gathering together editors and key contributors to highlight significant contributions of the past, present and future of genetics. This Topic, which comprises thirty rigorously reviewed manuscripts covering a wide spectrum of studies in humans, animals and plants, aims to give an overview of the most important areas and advances in genetics over the last 10 years, and to provide a platform to raise current challenges for exciting, new research.

The primary area of research within the Research Topic question how genes and genomes are related to phenotypes and human physiology. It includes research manuscripts and reviews that explore new etiology in human diseases, including, congenital hypopituitarism (https://doi.org/10.3389/fgene.2021.697549), *α* and *β*-Thalassemia in young children from regions in Southern China (https://doi.org/10.3389/fped.2021.724196), and the association between TNF- *α*-Polymorphisms and COPD susceptibility (https://doi.org/10.3389/fgene.2021.772032). Several manuscripts describe the value of different predictors in human disease, such as the value of longer telomere lengths in reducing risk of hip osteoarthritis (https://doi.org/10.3389/fgene.2021.718890), prognostic factors of lipid metabolism in obstructive sleep apnea (https://doi.org/10.3389/fgene.2021.747576), genetic analysis of coronary artery disease (https://doi.org/10.3389/fgene.2021.766485), use of integrated analysis of the RNA network in acute ischemic stroke (https://doi.org/10.3389/fgene.2022.833545), as well as the use of CLP1 as a prognostic biomarker of immune infiltrates in rheumatoid arthritis (https://doi.org/10.3389/fphar.2022.827215). The topic also includes a review on the role of copy number variation in autoimmune diseases (https://doi.org/10.3389/fgene.2021.794348).

Several original research articles describe the novel use of specific genes in diagnosis and prognosis of cancer, such as, ULBP1 in the case of adenocarcinoma (https://doi.org/10.3389/fgene.2022.762514), or use of enhancers as biomarkers of gastric cancer (https://doi.org/10.3389/fgene.2022.854211). A manuscript that describes the role of reduced mitochondrial content and immunocyte infiltration is also included in this topic (https://doi.org/10.3389/fgene.2022.832331). A review describes use of single-cell technologies and computational techniques for immune-profiling of the tumor environment (https://doi.org/10.3389/fgene.2022.867880). A case report describes identification of a novel mutation in AIMP2/P38, and its role in leukodystrophy, a progressive neurodevelopmental disorder (https://doi.org/10.3389/fgene.2022.816987). A second review article describes the role of homeobox genes in the genetic regulation of vertebrate forebrain development (https://doi.org/10.3389/fnins.2022.843794).

As another goal of Frontiers in Genetics is to support development of cutting edge technological and analytical tools to study genomic data, this topic also includes a review article that describes the role of genome-wide association studies (GWAS) in identification of thousands of single nucleotide polymorphism that have been associated with different human diseases and traits (https://doi.org/10.3389/fgene.2021.713230). In addition, an opinion article discusses the advantages and power of forming GWAS consortia to avoid some challenges associated with these studies and the cost of recruiting a large cohort (https://doi.org/10.3389/fgene.2021.801653). And another review discusses the role of molecular cytogenetics during highly used novel techniques such as chromosomics and cytogenomics analysis (https://doi.org/10.3389/fgene.2021.720507).

One of the new frontiers in the post-genomic era is three-dimensional (3D) genomics, which explores the relationship between chromatin spatial conformation and its effects on gene transcription. A review article of this topic describes the contribution of 3D organization in cancer biology (https://doi.org/10.3389/fcell.2022.879465). An additional review article discusses the role of the leucine-rich repeats containing G-protein coupled receptor 4 (LGR4) in energy metabolism, development and carcinogenesis https://doi.org/10.3389/fgene.2021.720507).

Another area that is covered in Frontiers in Genetics is gene flow among species and populations, including genomic research of farm animals. Here, we have included several manuscripts describing new findings on the role of genomics in animals, such as a review of longevity traits in Holstein Cattle (https://doi.org/10.3389/fgene.2021.695543), the molecular mechanisms of fat deposition in Xianyang Buffalo (https://doi.org/10.3389/fgene.2021.736441), and the study of variance components and heritability of semen traits over the reproductive life in boars (https://doi.org/10.3389/fgene.2022.805651). Adaptation to habitat and genetic diversity of the Silver Carp was also described (https://doi.org/10.3389/fevo.2022.850183), as well as a study of the role of evolutionary history in the formation of the regulatory regions of specific genes in different *Drosophila* species (https://doi.org/10.3389/fgene.2021.8072340.).

Interactions between organisms and their environments, as well as molecular and cellular genetics, are also covered in Frontiers in Genetics and in this Topic. An article describes an analysis of the regulation of DNA methylation during plant endosperm development (https://doi.org/10.3389/fgene.2022.760690). An additional manuscript describes the use of single-cell RNA-Seq and RNA-Seq to study the effects of 2,3,7,8-tetrachlorodibenzo-p-dioxin (TCDD) on zebrafish testes during sexual differentiation and its effects on sterility https://doi.org/10.3389/ftox.2022.821116.

There are number of cutting-edge technologies that will play an important part of future frontiers in genetics such as single-cell multiomics, new gene editing tool, e.g., Retron Library Recombineering (RLR) and others. This topic has several articles that focuses of these new frontiers, such as a review that discusses strategies to design high-efficiency mutations using the CRISPR/Cas system (https://doi.org/10.3389/fcell.2021.803252). Another review article describes the use of integrated and multi-omic approaches to study environmental and genomic interactions, and the impact of such interactions on human and animal health (https://doi.org/10.3389/fgene.2022.831866).

An interesting original study describes the use of micro RNA profiling in saliva to produce biomarkers of alcohol exposure in humans (https://doi.org/10.3389/fgene.2021.804222).

Frontiers would also like to take this opportunity to thank and congratulate the founding Field Chief Editor of Frontiers in Genetics, Professor, Dr. David B. Allison from Indiana University Bloomington, USA, who was leading the Field from 2010 until 2017. Dr. Allison’s vision and recognition of the value of Open Science directly resulted in establishing the journal’s worldwide reputation (Allison, 2011). This strong vision continued with Professor Emmanouil Dermitzakis, Ph.D., from University of Geneva Switzerland, the second Field Editor of Frontiers in Genetics, who served in this visionary role from 2017 until 2021. During his leadership, Frontiers in Genetics continued to expand the number of Specialties. In 2021, Professor Enrico Domenici, Ph.D., from University of Trento, Italy, became the third Field Chief Editor. Professor Domenici continues to successfully provide leadership and guidance in the further expansion of the field, https://www.frontiersin.org/journals/genetics/about.

Standing on the shoulders of giants, Frontiers in Genetics will continue to follow its mission and original vision to bring together the world’s leading experts in genetics to freely disseminate cutting-edge studies and accelerate the progress of research in all areas of genes and genomes in humans, plants, livestock and other model organisms. This also includes the increasingly pertinent ethical, legal and social implications of genomic studies that are also included in a section within Frontiers in Genetics.

The record of the first 10 years, and the continuation of truly wonderful experience, will move us further into the next decade and many more to come.
